# The impact of incidental durotomy on the outcome of decompression surgery in degenerative lumbar spinal canal stenosis: analysis of the Lumbar Spinal Outcome Study (LSOS) data—a Swiss prospective multi-center cohort study

**DOI:** 10.1186/s12891-016-1022-y

**Published:** 2016-04-18

**Authors:** Nils H. Ulrich, Jakob M. Burgstaller, Florian Brunner, François Porchet, Mazda Farshad, Giuseppe Pichierri, Johann Steurer, Ulrike Held, on behalf of the LSOS Study Group

**Affiliations:** Horten Centre for Patient Oriented Research and Knowledge Transfer, University of Zurich, Pestalozzistr. 24, 8091 Zürich, Switzerland; Spine Division, Balgrist University Hospital, University of Zurich, Zürich, Switzerland; Department of Orthopedics and Neurosurgery, Spine Center, Schulthess Clinic, Zurich, Switzerland

**Keywords:** Durotomy, Dural tear, Decompression, Degenerative lumbar spinal stenosis, Multi-center, Surgery

## Abstract

**Background:**

Incidental durotomy is a well-known complication during surgery for degenerative lumbar spinal stenosis (DLSS). In this prospective multicenter cohort study including eight medical centers our aim was to assess whether incidental durotomy during first-time lumbar spinal stenosis decompression surgery without fusion has an impact on long-term outcome.

**Methods:**

Patients of the multi-center Lumbar Stenosis Outcome Study (LSOS) with confirmed DLSS undergoing first-time decompression without fusion were enrolled in this study. Baseline patient characteristics and outcomes were analyzed at 6, 12, and 24 months follow-up respectively with the Spinal Stenosis Measure (SSM), the Numeric Rating Scale (NRS), Feeling Thermometer (FT), the EQ-5D-EL, and the Roland and Morris Disability Questionnaire (RMDQ).

**Results:**

A total of 167 patients met the inclusion criteria. Fifteen (9 %) of those patients had an incidental durotomy. Baseline characteristics were similar between the durotomy and no-durotomy group. All patients improved over time. In the group of durotomy patients, the median improvement in SSM symptoms scale was 1.1 points at 6 months, 1.1 points at 12 months, and 1.6 points at 24 months after baseline. For the no-durotomy group, these improvements were 0.8, 0.9, and 0.9. For SSM function the improvements were 1.0, 0.8, and 0.9 in the durotomy group, and 0.6, 0.8, and 0.8 in the no-durotomy group. None of the between-group differences were statistically significant.

**Conclusions:**

Incidental durotomy in patients with DLSS undergoing first-time decompression surgery without fusion did not have negative effect on long-term outcome and quality of life. However, only 15 patients were included in the durotomy group but these findings remained even after adjusting for observed differences in baseline characteristics.

## Background

Incidental durotomy is a potential complication of lumbar spinal surgery. Its incidence is reported to be up to 17 % depending on the literature reviewed [1–14]. Furthermore, it is well known that the type of surgical procedure performed, reoperations, and older age increase the risk of incidental durotomy [3–6, 9, 15, 16].

Persistent dural tears may cause various sequelae such as headache, meningeal pseudocyst formation, or dural cutaneous cerebrospinal fluid fistulas leading to meningitis and arachnoiditis [3, 12]. Recommendations to prevent these sequelae are primary repair, bed rest, and lumbar drain placement [3, 9, 14].

Currently, the impact of successfully treated dural tears on long-term outcomes is still controversial. One publication issued in 2005 demonstrated an association with poor clinical outcome in patients undergoing lumbar disc surgery [12] whereas other studies found no long-term sequelae in patients undergoing spinal surgery [3, 6, 9, 17]. However, long-term effects for decompression surgery only without fusion specifically for degenerative lumbar spinal stenosis (DLSS) have not been studied so far. Lumbar spinal stenosis is the most frequent indication for spine surgery in patients older than 65 years [18]. For instance, in the metropolitan area of Zurich with around 1.3 million inhabitants almost 1000 lumbar decompression surgeries without fusions in patients with DLSS are performed every year [19].

The purpose of this study was to assess whether incidental durotomy during first-time decompression surgery without fusion for degenerative lumbar spinal stenosis had an impact on long-term outcomes. We used data from the Lumbar Stenosis Outcome Study (LSOS) [20], a multi-center prospective cohort study, to explore this issue.

## Methods

### Patient selection

Patients with a history of neurogenic claudication were recruited from outpatient clinics at all participating centers. Eligible patients had no evidence of stenosis caused by tumor, fracture, infection, or significant deformity (>15° lumbar scoliosis), and were aged 50 years or more. Magnetic Resonance Imaging (MRI) verified lumbar spinal canal stenosis. None of the patients had prior lumbar spine surgery. Furthermore, patients had no clinical peripheral artery occlusive disease (confirmed by a vascular specialist in patients without palpable pulses in the lower limb).

### Surgical procedure

Surgery consisted of a standard open posterior lumbar laminectomy or laminotomy at the affected level or levels without instrumentation. Decompression of the lateral recessus and foramina was performed when necessary to decompress the local nerve roots.

### Radiological classification

The MRI of each patient was evaluated by a senior radiologist. He categorized the severity of the stenosis (central, lateral recess, and neural foraminal) of each level into “no”, “mild”, “moderate”, or “severe” according to the consensus paper on core radiological parameters of the LSOS-study [21].

### Data collection and follow-up

Parts of the basic data sheet were interview-administered and recorded by a study coordinator. All other questionnaires were self-administered and completed by the patients themselves. All data were collected at baseline, and at 6 months. Long-term outcome data was gathered after 1 and 2 years.

### Questionnaires

#### Spinal Stenosis Measure (SSM)

The SSM, an instrument specifically developed for spinal stenosis patients by Stucki et al. [22], targets to measure severity of symptoms and quantifies disability of the lumbar spinal stenosis population. It is recommended by the North American Spine Society (NASS) and used in different studies on lumbar spinal stenosis [23–26]. It consists of three different subscales; *the symptom severity subscale*, *the physical function subscale* and the *satisfaction subscale*. The symptom severity scale can be divided into a pain domain (severity, frequency and back pain) and a neuroischemic domain (leg pain, weakness, numbness and balance disturbance). Score range is from 1 to 5 and 1 to 4 (best-worst).

#### Feeling Thermometer (FT) and Numeric Rating Scale (NRS)

General assessment of lumbar spinal stenosis symptoms such as lower extremity pain and discomfort are measured. Score range is from 0 to 100 and 0 to 10 (best-worst).

*EQ-5D-3L:* The EQ-5D-3L is an assessment tool to measure health-related quality of life. It measures general non-disease specific health-related quality of life, including physical, mental and social dimensions [27]. The health status measures five dimensions of health (*mobility, self-care, usual activities, pain/discomfort and anxietycpdepression*) which can be calculated as a sum score (score range 0–100, worst-best) [27]. The second part of the questionnaire estimates patient’s *actual health* status (score range 0–100, worst-best).

#### Roland and Morris Disability Questionnaire (RMDQ)

The Roland and Morris Disability Questionnaire is a back pain specific, self-rated physical disability questionnaire developed by Roland and Morris in 1983 [28]. Disability is measured respective to the following categories: physical function activities and activities of daily living including eating and sleeping. Score range is from 0 to 24 (best-worst).

#### Cumulative Illness Rating Scale (CIRS)

Comorbidity is measured using CIRS that rates the presence and severity of comorbid diseases in 14 organ systems (according to modified version by Miller et al. [29]). Score range is from 0 to 56 (best-worst).

#### Hospital Anxiety and Depression Scale (HADS)

The HADS was originally developed to measure anxiety and depression in a hospital setting [30], however, it is nowadays common to use it in all settings [31]. It contains two 7-item subscales for anxiety and depression with a score range of 0–21 (best-worst) each.

### Outcomes

Main outcomes were changes in SSM symptoms and function, NRS, FT, EQ-5D-EL sum score and actual health status, and RMDQ between the durotomy and no-durotomy group.

### Statistical analyses

Analysis of data consisted of descriptive statistics of patient demographics and outcomes. Continuous variables were shown as median and interquartile ranges and categorical variables were shown as numbers and percentages of total. To evaluate differences between patients of the durotomy and no-durotomy groups, we used Wilcoxon tests for the continuous variables and chi-squared tests for the categorical variables. We calculated changes from baseline at 6 months, 12 months, and 24 months for the main outcome variables SSM symptoms and function, NRS, FT, EQ-5D-EL sum score and actual health status, and RMDQ. To assess whether these changes differed significantly across the durotomy versus no-durotomy group, we used Wilcoxon tests. In order to reduce the probability of false positive findings, we used Bonferroni adjustment of the global significance level α = 0.05. The number of statistical tests was 50, therefore the new significance level α* = 0.05/50 = 0.001. If there are differences between the baseline characteristics in the durotomy and the no-durotomy groups, we will adjust for these baseline characteristics for changes in long term outcomes using multiple linear regression models with durotomy (yes/no) as the determinant.

For graphical representations of SSM symptoms and function, and EQ-5D-EL sum score and actual health status over time, boxplots were used.

All analyses were conducted with R for Windows [32].

## Results

### Patient characteristics

Table 1 presents the patients characteristics at baseline. A total of 167 patients met the inclusion criteria; in 15 of these, a durotomy occurred (prevalence: 9 %). There were no significant differences in any of the baseline characteristics. Overall, 88 of 167 patients (52.7 %) were female, median age was 75 years (IQR 12), and median body mass index was 26.3 kg/m^2^ (IQR 6.1). Of the study population 104 (62.3 %) patients hold higher education degree (no university) and 27 (16.2 %) hold a university degree. Seven patients (46.7 %) had previous lumbar epidural steroid injections in the durotomy group, and 84 patients (55.3 %) in the no-durotomy group. There were also no statistically significant differences in all questionnaires.

**Table 1:** Patient characteristics

Characteristics	Durotomy (*n* = 15)	No durotomy (*n* = 152)	*p*
Age, median (IQR), y	73 (11)	75 (12)	0.61
Female, n (%)	6 (40)	82 (53.9)	0.45
BMI, median (IQR), kg/m2	26 (8.2)	26.3 (5.8)	0.82
Diabetes mellitus, n (%)	16 (10.5)	2 (13.3)	0.99
Smoker, n (%)	1 (6.7)	13 (8.6)	0.69
Level of education			0.10
Compulsory education (1–9 years), *n* (%)	4 (26.7)	32 (21.1)	
Higher education/vocational training (no university) (10–12 years), *n* (%)	6 (40)	98 (64.5)	
University degree, *n* (%)	5 (33.3)	22 (14.5)	
CIRS, median (IQR)	7 (5.5)	9 (4.5)	0.15
HADS anxiety, median (IQR)	2 (3.5)	4 (4)	0.06
HADS depression, median (IQR)	3 (3)	4 (4.5)	0.13
SSM symptoms, median (IQR)	3.1 (0.5)	3.1 (1)	0.84
SSM functions, median (IQR)	2 (0.8)	2.4 (1)	0.21
NRS, median (IQR)	6 (1.5)	7 (3)	0.76
Feeling thermometer, median (IQR)	70 (22.5)	70 (30)	0.90
EQ-5D-EL sum score, median (IQR)	70 (25)	70 (20)	0.99
EQ-5D-EL actual health status, median (IQR)	80 (34)	65 (40)	0.19
RMDQ, median (IQR)	14 (8)	13 (8)	0.64
Prior lumbar epidural steroid injection, n (%)	7 (46.7)	84 (55.3)	0.71

*BMI* body mass index, *CIRS* cumulative illness rating scale, *FT* feeling thermometer, *HADS* hospital anxiety and depression scale, *IQR* interquartile range, *NRS* numeric rating scale (NRS), *RMDQ* Roland and Morris disability questionnaire, *SSM* spinal stenosis measure

### Surgical characteristics

There were no significant differences between the durotomy and no-durotomy group in decompression levels, and in the number of decompressed levels. In both groups, around 80 % of the patients were operated microscopically (Table 2).

**Table 2:** Comparison of perioperative outcomes and radiological parameters between the durotomy and no-durotomy group

Outcome	Durotomy (*n* = 15)	No durotomy (*n* = 152)	*p*
Decompression level, n (%)			
L1/L2	1 (6.7)	4 (2.6)	0.94
L2/L3	6 (40.0)	35 (23.0)	0.25
L3/L4	10 (66.7)	99 (65.1)	0.99
L4/L5	11 (73.3)	126 (82.9)	0.57
L5/S1	2 (13.3)	27 (17.8)	0.94
Levels decompressed, n (%)			0.49
1	5 (33.3)	50 (32.9)	
2	5 (33.3)	70 (46.1)	
≥ 3	5 (33.3)	32 (21.1)	
OP technique, n (%)			0.90
Conventional	2 (13.3)	23 (15.1)	
Microscopic	12 (80.0)	123 (80.9)	
No. of severe stenotic levels, n (%)			0.57
0^a^	1 (6.7)	11 (7.2)	
1	4 (26.7)	48 (31.6)	
2	4 (26.7)	57 (37.5)	
≥ 3	6 (40.0)	36 (23.7)	
No. of mild/moderate stenotic levels, n (%)			0.63
0^b^	1 (6.7)	5 (3.2)	
1	2 (13.3)	13 (8.6)	
2	5 (33.3)	38 (25.0)	
≥ 3	7 (46.7)	96 (63.2)	

^a^No severe stenotic levels

^b^Only severe stenotic levels

40.0 % of the patients in the durotomy and 23.7 % in the no-durotomy group had three or more severe stenotic levels whereas 46.7 and 63.2 %, respectively, had three or more mild or moderate stenotic levels. Neither was statistically significant.

### Surgical management of durotomy

All 15 patients experienced incidental durotomy during surgery were successfully treated intraoperatively. In nine patients (60 %) the durotomy was firstly sutured, afterwards either a patch and/or glue was applied. Two patients (13 %) only received a patch, in one patient (7 %) only glue was applied, and in the remaining three patients (20 %) a patch and glue were applied. No lumbar drain was placed.

### Intra- and postoperative complications

Only one patient (0.7 %) in the no-durotomy group experienced a prominent epidural bleeding due to a large epidural venous plexus during surgery, that led to revision surgery on the next day (Table 3). Postoperative wound infection occurred in one patient (6.7 %) in the durotomy group, and in two patients (1.3 %) in the no-durotomy group. Other postoperative complications (e.g., urosepsis, hemorrhage, wound healing deficit) were seen in 20.0 and 10.5 % of the patients, respectively. None of these differences were statistically significant. Furthermore, no patient died within 3 months postoperatively.

**Table 3:** Intra- and postoperative complications

Outcome	Durotomy (*n* = 15)	No durotomy (*n* = 152)	*p*
Intraoperative complications, n (%)			
Epidural venous bleeding	0 (0)	1 (0.7)	0.99
Other	0 (0)	0 (0)	
None	15 (100)	151 (99.3)	
Postoperative complications, *n* (%)			
Wound infection	1 (6.7)	2 (1.3)	0.60
Other	3 (20.0)	16 (10.5)	0.99
None	11 (73.3)	134 (88.2)	
postoperative mortality (death within 6 weeks of surgery), n (%)	0 (0)	0 (0)	
postoperative mortality (death within 3 months of surgery), n (%)	0 (0)	0 (0)	

### Changes in main outcomes from baseline to 6 months, 12 months, and 24 months

All patients improved over time. In the group of durotomy patients, the median improvement was 1.1 points at 6 months, 1.1 points at 12 months, and 1.6 points at 24 months after baseline in SSM symptoms scale. For the no-durotomy group, these improvements were 0.8, 0.9, and 0.9. For SSM function the improvements were 1.0, 0.8, and 0.9 in the durotomy group, and 0.6, 0.8, and 0.8 in the no-durotomy group. The corresponding changes over time for the other questionnaires can be found in Table 4. None of the group differences between the no-durotomy and the durotomy group were statistically significant. Lack of statistically significant differences in the baseline characteristics could be due to the unequal size of the durotomy and the no-durotomy groups. Studying the baseline characteristics apart from *p*-values, the variables age, gender, higher education, HADS anxiety, and EQ-5D-EL actual health status seemed to make the groups not directly comparable. Therefore, we fitted multiple linear regression models to the long term outcomes, and estimated the effect of durotomy, adjusted for the above mentioned variables simultaneously. It turned out that the estimated adjusted effects were of the same size as the unadjusted changes presented in Table 4, and none of these were statistically significant either.

**Table 4:** Changes in main outcomes from baseline to 6, 12, and 24 months: median (IQR)

Outcome	Baseline – 6 months			Baseline – 12 months			Baseline – 24 months		
	Durotomy	No durotomy	*p*	Durotomy	No durotomy	*p*	Durotomy	No durotomy	*p*
∆ SSM symptoms	1.1 (0.9)	0.8 (1.3)	0.02	1.1 (0.8)	0.9 (1)	0.19	1.6 (0.9)	0.9 (1)	0.13
∆ SSM function	1 (0.4)	0.6 (1)	0.12	0.8 (0.6)	0.8 (1)	0.53	0.9 (0.6)	0.8 (1.1)	0.48
∆ NRS	4 (2.5)	3 (4)	0.13	4 (2.5)	3 (4)	0.24	5 (1.8)	4 (4.2)	0.22
∆ FT	45 (28.5)	30 (40)	0.08	45 (29)	34.5 (48.2)	0.2	60 (16.2)	34 (39.5)	0.06
∆ EQ-5D-EL sum score	−20 (25)	−10 (30)	0.04	−20 (20)	−20 (30)	0.17	−20 (17.5)	−15 (30)	0.45
∆ EQ-5D-EL ahs	−9 (29.5)	−10 (33.5)	0.97	−5.5 (28.8)	−10 (37.5)	0.83	−6.5 (14)	−10 (35.5)	0.42
∆ RMDQ	6 (7)	3 (6)	0.03	8 (5)	4 (7)	0.01	9.5 (9)	4 (8)	0.02

*Ahs* actual health status, *FT* feeling thermometer, *NRS* numeric rating scale (NRS), *RMDQ* Roland and Morris disability questionnaire, *SSM* spinal stenosis measure

Numbers of patients: Baseline – 6 months: 15 patients in the durotomy group, 152 patients in the no durotomy group. No missing values. Baseline – 12 months, 15 patients in the durotomy group, 152 patients in the no durotomy group. No missing values in SSM subscales, single missing values in some secondary outcomes. Baseline – 24 months, 11 patients in the durotomy group, 90 patients in the no durotomy group for SSM subscales. Single missing values in some outcomes

However, the durotomy group experienced greater improvements in all scales at all follow-up points except in the EQ-5D-EL actual heath status than the no-durotomy group.

**Fig. 1:**
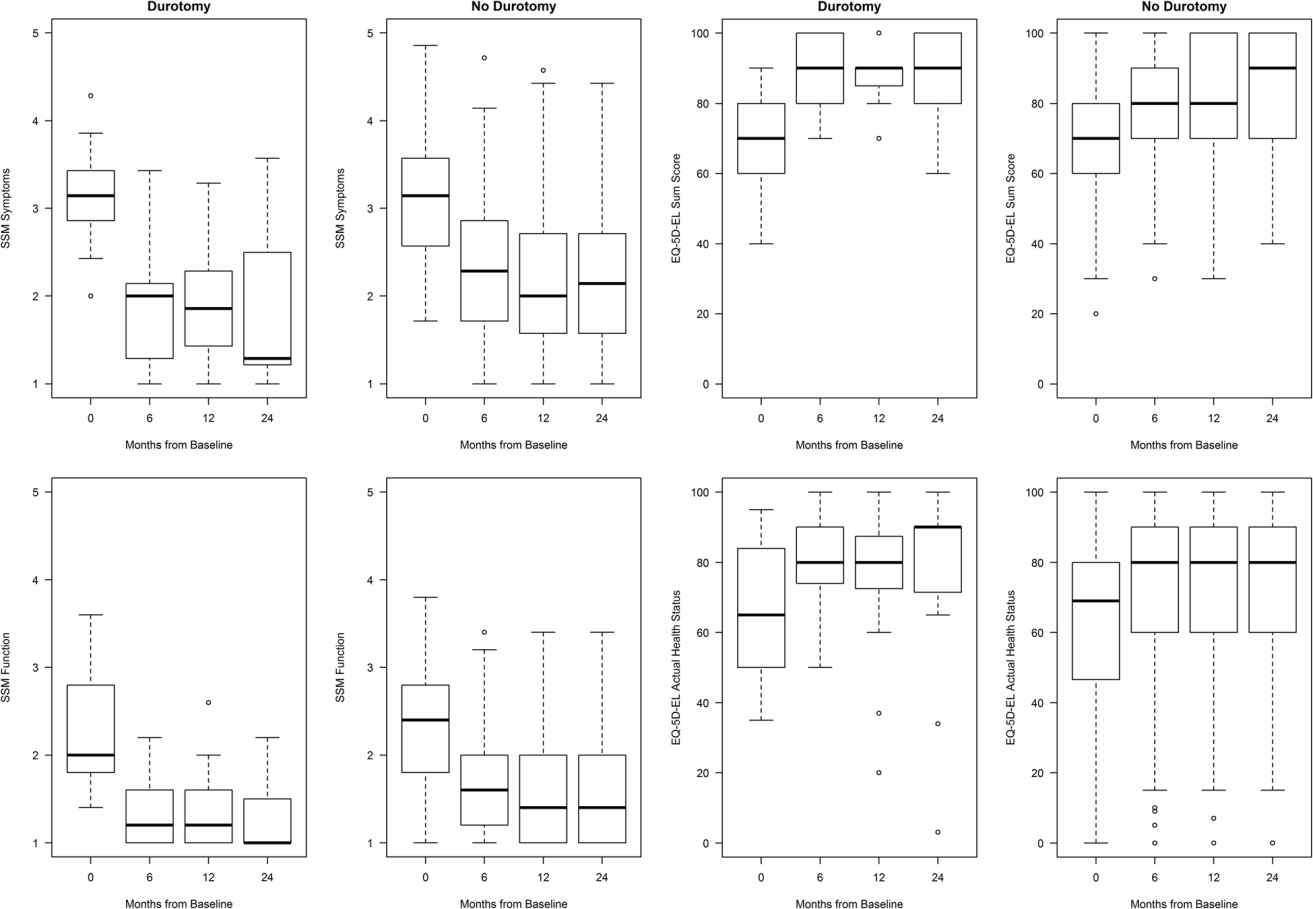
SSM symptoms and function and EQ-5D-EL at baseline, 6, 12, and 24 months (durotomy/no-durotomy group)

Figure 1 shows SSM symptoms and function, EQ-5D-EL sum score and actual health status over time with boxplots.

## Discussion

In this study we investigated 167 consecutive patients with symptomatic degenerative lumbar spinal canal stenosis undergoing lumbar decompression surgery without fusion. Of these, 15 patients (9 %) experienced incidental durotomy during surgery. We assessed changes in the outcomes Spinal Stenosis Measure (SSM), the Numeric Rating Scale (NRS), Feeling Thermometer (FT), the EQ-5D-EL, and the Roland and Morris Disability Questionnaire (RMDQ) over time. Patients with and without incidental durotomy improved from baseline to 24 months of follow-up. Comparing the improvements between the groups, we found greater improvement in the durotomy-group but the difference was not statistically significant. The results of our study indicate that incidental durotomy followed by appropriate repair measures has no negative effect on the above described outcomes.

Other studies analyzed the impact of incidental durotomy on outcomes and reported conflicting results. Desai et al. [17] showed that dural tears did not have any negative impact on outcome in their multi-center study with 409 patients and 37 cases of incidental durotomy. Wang et al. [9] reported in a series of 88 patients with durotomy that incidental durotomy did not compromise long-term outcome if treated appropriately. Furthermore, Grannum et al. [10] demonstrated in a case-control study that incidental durotomy was not associated with less improvement over time. Recent data from Stromqvist et al. [33] showed that dural tears did not compromise the results of discectomy at 1 year post-surgery. Cammisa et al. [3] came to similar conclusions in their study of 66 patients with incidental durotomies. All previous studies were based on retrospectively collected data, except the publications by Desai et al. [17] and Grannum et al. [10] Furthermore, they included patients who underwent different spine procedures such as lumbar discectomies, fusion surgeries, and revision surgeries.

In contrast to the above mentioned studies, Saxler et al. [12] reported a tendency for persisting back pain, higher number of re-operations, and longer duration of inability to work in a group of 41 patients with incidental durotomy after lumbar discectomy as compared with 41 appropriately matched patients without dural tears. These results are not easily comparable to our findings because the indication for surgery was quite different.

The prevalence of dural tears in our study was 9 %. This rate is comparable to that reported for lumbar spine surgery in other large series [3, 6, 9, 14, 17, 34]. Different patient characteristics like gender, age and severity of stenosis, as well as experience of the surgeon and surgical procedure may attribute to a certain variation across studies (1–17 %).

Most common complications associated with durotomies include headache, wound infection, meningeal pseudocyst formation, or dural cutaneous cerebrospinal fluid fistulas leading to meningitis and arachnoiditis [3, 12]. In the present study we did not observe a higher incidence of intraoperative vascular injury, or postoperative wound infection compared to other studies [3, 10, 17]. Furthermore, no neurological complications, cerebrospinal fluid fistula formation, or other surgical complications occurred.

Our study has several strengths. These include the multi-center setting and prospective collection of data, as well as the use of established questionnaires on degenerative lumbar spinal stenosis. Four out of eight study centers are teaching hospitals in which residents assist spine surgeries. Nevertheless, our durotomy prevalence rate was within the range of other studies. Furthermore, our patient sample was very homogenous since only first-time decompression surgeries without fusion were included. In addition to that, the groups of patients with and without incidental durotomy were comparable with respect to their baseline characteristics.

A limitation of our study was the restricted number of 15 patients with durotomy. A higher number of patients with a durotomy would be desirable for future investigations. Furthermore, we did not collect detailed information on the occurrence and duration of postoperative headache, and pseudomeningocele. These are typical complications associated with dural tears [35, 36]. Postoperative management of incidental durotomy often involves placement of a subarachnoid drain and postoperative bed rest, which can lead to extended hospital stay. The recording of specific treatment modalities and the length of hospital stay was not part of LSOS-study and should be included in future studies.

An unexpected finding of our study was the tendency for better outcomes in the durotomy group as compared to the no-durotomy group. Several other studies investigated preoperative predictors for clinical outcome after lumbar spinal stenosis surgery. Aalto et al. [37] found that depression, cardiovascular comorbidity, and reduced walking ability caused by disorders had an adverse influence on patients’ subjective outcomes. In another review article by McKillop et al. [38], preoperative depression was associated with postoperative symptom severity and disability. In our patient sample, the durotomy group had a lower preoperative HADS depression score that could have influenced the better but not statistically significant postoperative outcome in contrast to the no-durotomy group. Another reason for greater improvement in the durotomy group could be the higher number of severe stenotic levels (40 %) in comparison to the no-durotomy group (23 %). Decompression surgery might have been more difficult in patients with a higher number of severe stenotic levels, and thus might increase the probability for dural tears. On the other hand, these patients were more likely to benefit from surgery that was ultimately reflected by our outcome measures.

## Conclusions

Incidental durotomy in patients with degenerative lumbar spinal stenosis undergoing first-time decompression surgery without fusion did not have negative effect on long-term outcome and quality of life. However, only 15 patients were included in the durotomy group but these findings remained even after adjusting for observed differences in baseline characteristics.

## Ethics

This multi-center cohort study was conducted in compliance with all international laws and regulations as well as any applicable guidelines. Written informed consent to participate in the study has been obtained from participants. The study was approved by the independent Ethics Committee of the Canton Zurich (KEK-ZH-NR: 2010-0395/0).

## Availability of data and materials

Materials described in the manuscript, including all relevant raw data, is not freely available because LSOS is an ongoing study. All data will be freely available after follow-up time in 2021.

## Abbreviations

CIRS: cumulative illness rating scale
DLSS: degenerative lumbar spinal stenosis
FT: feeling thermometer
HADS: hospital anxiety and depression scale
IQR: interquartile range
LSOS: lumbar stenosis outcome study
LSS: lumbar spinal stenosis
MRI: magnetic resonance imaging
NASS: north american spine society
nrs: the numeric rating scale
RMDQ: roland and morris disability questionnaire
SSM: spinal stenosis measure
